# Evaluation of Hydrogel Suppositories for Delivery of 5-Aminolevulinic Acid and Hematoporphyrin Monomethyl Ether to Rectal Tumors

**DOI:** 10.3390/molecules21101347

**Published:** 2016-10-12

**Authors:** Xuying Ye, Huijuan Yin, Yu Lu, Haixia Zhang, Han Wang

**Affiliations:** 1 Cardiovascular Medicine, Tianjin First Center Hospital, Tianjin 300192, China; l-long@126.com; 2 Laser Medicine Laboratory, Institute of Biomedical Engineering, Chinese Academy of Medical Sciences & Peking Union Medical College, Tianjin 300192, China; luyu507@aliyun.com (Y.L.); hbziqing@163.com (H.Z.); wanghan87816@126.com (H.W.)

**Keywords:** photosensitizer, drug delivery, photodynamic therapy, hematoporphyrin monomethyl ether, 5-aminolevulinic acid, rectal cancer

## Abstract

We evaluated the potential utility of hydrogels for delivery of the photosensitizing agents 5-aminolevulinic acid (ALA) and hematoporphyrin monomethyl ether (HMME) to rectal tumors. Hydrogel suppositories containing ALA or HMME were administered to the rectal cavity of BALB/c mice bearing subcutaneous tumors of SW837 rectal carcinoma cells. For comparison, ALA and HMME were also administered by three common photosensitizer delivery routes; local administration to the skin and intratumoral or intravenous injection. The concentration of ALA-induced protoporphyrin IX or HMME in the rectal wall, skin, and subcutaneous tumor was measured by fluorescence spectrophotometry, and their distribution in vertical sections of the tumor was measured using a fluorescence spectroscopy system. The concentration of ALA-induced protoporphyrin IX in the rectal wall after local administration of suppositories to the rectal cavity was 9.76-fold (1 h) and 5.8-fold (3 h) higher than in the skin after cutaneous administration. The maximal depth of ALA penetration in the tumor was ~3–6 mm at 2 h after cutaneous administration. Much lower levels of HMME were observed in the rectal wall after administration as a hydrogel suppository, and the maximal depth of tumor penetration was <2 mm after cutaneous administration. These data show that ALA more readily penetrates the mucosal barrier than the skin. Administration of ALA as an intrarectal hydrogel suppository is thus a potential delivery route for photodynamic therapy of rectal cancer.

## 1. Introduction

Photodynamic therapy (PDT) is a clinical treatment that combines visible light irradiation and a photosensitizing drug to cause destruction of selected cells [1]. To date, PDT has been primarily investigated as a treatment for tumors and neoplasias of the skin, bladder, head and neck, and female reproductive tract, and for treatment of non-neoplastic diseases such as psoriasis, age-related macular degeneration of the eye, and microbiological infections of skin wounds, burns, and ulcers [2,3,4,5,6].

PDT is especially well suited for epithelial tumors that are easy to irradiate with visible light. However, thanks to the development of fiber optic-based interstitial, intravesical, and endoscopic light delivery systems [7,8,9], it is now possible to treat basal and parenchymal lesions of virtually any part of the body [1].

Colorectal cancer (CRC) is the third most common cancer in men (10% of total cancer incidence) and the second in women (9.2%) worldwide [10]. The high prevalence, invasiveness, metastatic potential, and probability of recurrence of CRC suggest the need for radical treatment methods that selectively target cancer cells [11]. PDT has several attributes consistent with this need: high tumor selectivity, lack of cross-resistance, a wide range of total light and drug dose that allow multiple applications of PDT with the same tumor, and a very good cosmetic effect, with little or no scarring [12]. Despite very promising performances in preclinical and clinical studies of CRC, PDT is still not considered in the treatment guidelines [13,14]. In the absence of precise guidelines for the use of PDT in patients with CRC, a large variety of PDT parameters, including the photosensitizer used, its concentration, the type and the dosage of light, and the application regimen, must be taken into account when PDT is performed in the clinic [15,16,17]. Photosensitizers (PS) such as porphyrins, chlorins, phthalocyanines, texaphyrins, and 5-aminolevulinic acid (ALA), delivered intravenously or orally, have been investigated for the treatment of CRC in animal models and in the clinic. In addition, new photosensitizing molecules have been developed and novel modifications of known photosensitizers have been studied to enhance the specific uptake by targeted CRC cells [18,19,20]. However, little research has been carried out on development innovative formulations for photosensitizer delivery.

As delivery vehicles to the rectosigmoid region, suppositories exhibit prolonged retention, more uniform coating of the mucosa, and better acceptance by the patient compared with other formulations [21]. In this study, we evaluated the uptake efficiency of hydrogel-associated ALA and hematoporphyrin monomethyl ether (HMME) delivered topically to rectal tumors. We compared intrarectal delivery with the most commonly used delivery routes in an effort to find a more effective route for currently used photosensitizers. Our goal is to improve PDT efficiency for rectal tumors and thus accelerate its use in clinical practice.

## 2. Results

### 2.1. Release Studies of PS Hydrogel in Vitro

**Figure 1:**
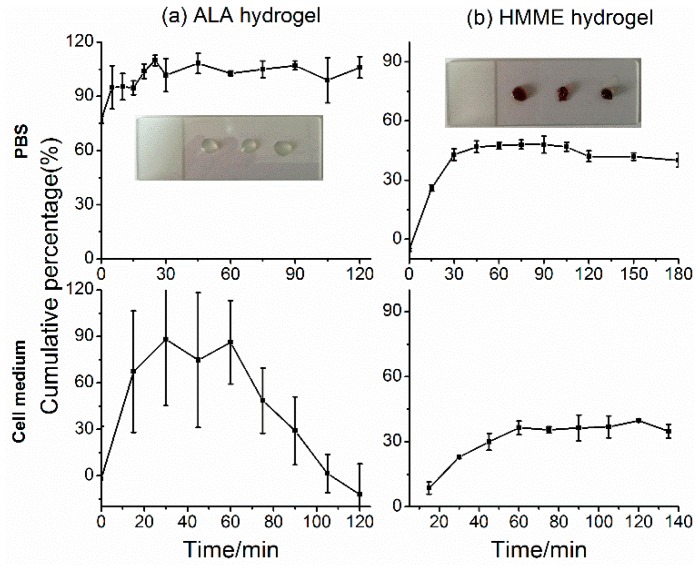
Release studies of photosensitizers (PS) hydrogel in vitro. (**a**) 5-aminolevulinic acid (ALA); (**b**) Hematoporphyrin monomethyl ether (HMME). The upper row refers to the release of PS hydrogel in PBS, and the lower row indicates the release of PS hydrogel in RPMI1640 medium with SW837 cells cultured. The inserts show the appearance of the formed PS hydrogel.

ALA and HMME hydrogel were produced by the set of alginate-CaCl_2_ shown as the inserts in Figure 1. ALA released completely from the hydrogel in quite a short time 10 min in PBS and 30 min in RPMI1640 medium with cultured cells (Figure 1a,b). The cumulative percentage dropped down from 60 min implied that the released ALA was quickly taken up by the cells (Figure 1b). As a comparison, HMME released much slower than ALA both in PBS and cell medium but still began to release in 15 min. In PBS, just about half of the amount of HMME released from the hydrogel after 8 h and the cumulative percentage decreased from 2 h was due to the removal of HMME for detection. In cell medium, the percentage of HMME concentration remained around 36.8% which hinted the balance between HMME release and uptake by cells.

### 2.2. Standard Curves of PS in Vivo

Mouse plasma was used to construct standard curves of HMME and protoporphyrin IX (PpIX), which is a fluorescent product of intracellular ALA metabolism. The standard curves for both compounds were linear over the range 0.01–1.0 µg/mL. The regression coefficient (r-squared) was 0.99952 for HMME and 0.9884 for PpIX (Figure 1). The linear equations for HMME is

*y* = 893.77414*x* + 0.31118 (1)

and for PpIX (ALA) is

*y* = 373.7246*x* − 0.09975 (2)

where *x* represents the photosensitizer concentration and y represents the relative fluorescence intensity detected.

**Figure 2:**
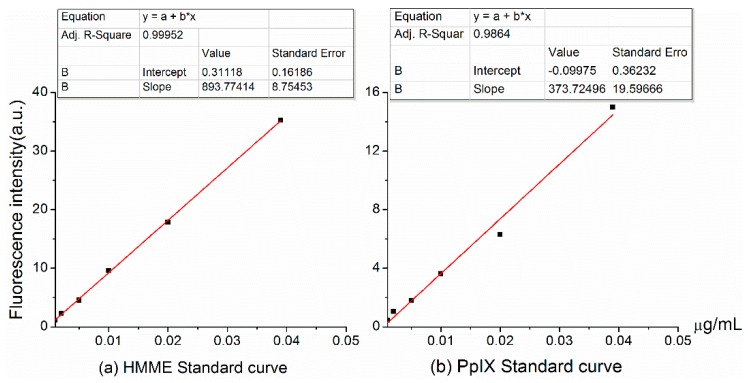
Standard curves of photosensitizer concentrations. (**a**) Hematoporphyrin monomethyl ether; (**b**) protoporphyrin IX.

The linear equations derived here (shown as Figure 2) were used to determine the amount of photosensitizer in the experimental tissue samples by factoring in the solution volumes and dilutions and the original weight of the tissue. This method was used to calculate the photosensitizer concentrations presented in the following sections.

### 2.3. Uptake of Photosensitizers in the Rectal Wall

**Figure 3:**
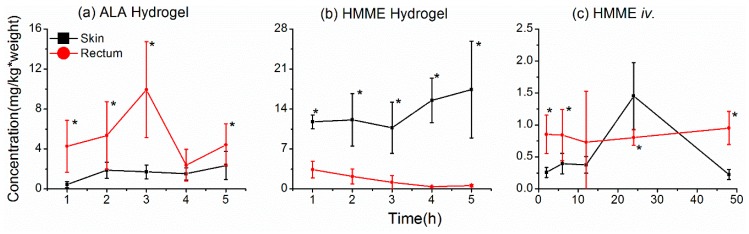
Uptake of photosensitizers into the rectal wall and skin. (**a**,**b**) Levels of 5-aminolevulinic acid (ALA) (**a**) or hematoporphyrin monomethyl ether (HMME) (**b**) were measured in the skin or rectum after cutaneous or intrarectal administration of a hydrogel, respectively; (**c**) levels of HMME were measured in the skin and rectum after intravenous injection. Photosensitizer concentrations are represented as mg/kg of tissue. The asterisks * mean significant difference (*p* < 0.05) between the two tissues at the same time point.

We first compared the uptake of ALA and HMME in the rectal wall and skin after local application (intrarectal or cutaneous, respectively). Hydrogels containing ALA (20% *w*/*v*) or HMME (1 mg/mL) were administered intrarectally or applied directly to the skin overlying ectopic tumors formed by subcutaneous injection of SW837 human rectal carcinoma cells. At various times thereafter, the photosensitizer concentrations in the rectal wall and skin were measured. Uptake of ALA into the rectal wall was much greater than in the skin after local application to either site. Indeed, levels of PpIX were 9.76-fold and 5.8-fold higher in the rectal wall than in the skin at 1 h and 3 h after administration, respectively (Figure 3a). The uptake of ALA in the rectal wall reached a peak at 3 h and decreased thereafter, whereas ALA levels in the skin were still rising at 5 h after administration. These data imply that ALA delivered by hydrogel suppository accumulated more rapidly and to greater levels in rectal wall than it did in the skin after cutaneous delivery. In contrast, there was little uptake of HMME in the rectal wall, and the level decreased steadily over time, whereas HMME levels in the skin were maintained at relatively high levels during the same time frame following cutaneous application (Figure 3b). It is possible that dermal absorption of HMME is hampered by its high molecular weight [18] and that the relatively high concentrations of HMME in the skin might reflect its persistence there, whereas HMME administered intrarectally would most likely be removed by mucus secreted by the rectum. We also examined the uptake of HMME in the rectal wall and skin after intravenous administration (Figure 3c). Here too, we observed higher concentrations of HMME in the rectal wall than in the skin, except for a sharp increase in skin levels at 24 h after injection. These data indicate that HMME is more efficiently delivered to the rectal wall by intravenous injection than by local application as a hydrogel suppository.

### 2.4. Uptake of Photosensitizers by Rectal Tumors

**Figure 4:**
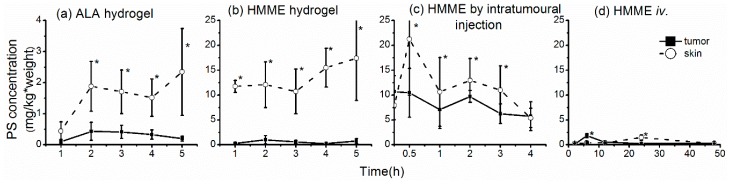
Concentrations of photosensitizer in subcutaneous tumors and skin after administration by various routes. (**a**,**b**) Levels of 5-aminolevulinic acid (**a**) and hematoporphyrin monomethyl ether (HMME) (**b**) were measured in tumor and skin samples after application of hydrogels to the skin overlying the tumor; (**c**,**d**) HMME levels were measured in tumor and skin samples after injection in phosphate-buffered saline intratumorally (**c**) or intravenously (**d**). Photosensitizer concentrations are presented as mg/kg of tissue. The asterisks * mean significant difference (*p* < 0.05) between the two tissues at the same time point.

Our aim is to determine the extent to which photosensitizers are taken up by rectal tumors; however, there are considerable technical challenges associated with modeling rectal tumors in situ. Therefore, we measured photosensitizer uptake in the skin, rectal wall, and subcutaneous tumors and then used that information to estimate the uptake by rectal tumors in situ. When ALA hydrogel was applied to the skin, the level of ALA-derived PpIX in the underlying tumor increased within 2 h and remained at relatively high levels for up to 4 h (Figure 4a). However, application of HMME in hydrogel form led to very little uptake by the tumor, even though a high concentration of HMME was present in the skin (Figure 4b), which likely reflects its adherence to the skin. As expected, high levels of HMME were seen in tumors after intratumoral injection; however, the skin also contained high HMME concentrations, perhaps because of leakage from the needle during entry or withdrawal (Figure 4c). After intravenous injection of HMME, peak uptake in the skin was observed at 24 h and in the tumor at 6 h (Figure 4d). This large difference between the time to peak HMME uptake by the tumor and skin suggests that PDT could be applied specifically to the tumor at 6 h after HMME injection, at which time HMME uptake by the tumor is at a peak.

**Figure 5:**
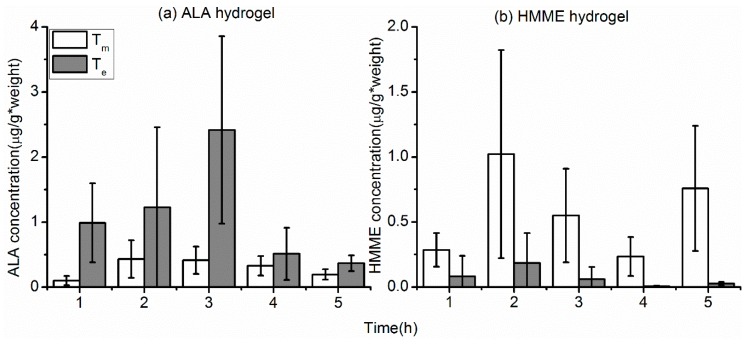
Estimated concentrations of 5-aminolevulinic acid (ALA) (**a**) and hematoporphyrin monomethyl ether (HMME) (**b**) in rectal tumors based on modeling of subcutaneous tumors. T_m_ is the measured concentration of ALA in the subcutaneous tumors and T_e_ is the estimated concentration of ALA in a rectal tumor.

We applied these findings to the formula

(3) $$T_{e}=R_{m}\times \frac{T_{m}}{S_{m}}$$

where T_e_ is the estimated concentration of ALA or HMME in the rectal tumor in situ, R_m_ is the measured concentration of PS in the rectal wall, T_m_ is the measured concentration of PS in the subcutaneous tumor, and S_m_ is the measured concentration of PS in the skin. Using this formula, we estimated that the concentration of ALA in the rectal tumor would be 3–5 times greater than that measured in the subcutaneous tumor, depending on the time after administration (Figure 5a). This suggests that local administration in the form of a suppository could be an effective route for delivery of ALA to rectal tumors. However, HMME was found hard to penetrate the skin and mucosa barrier (Figure 3b and Figure 4b) because of its high molecular weight. The same results were also confirmed by the formula (Figure 5b).

### 2.5. ALA Penetration into Tumors

**Figure 6:**
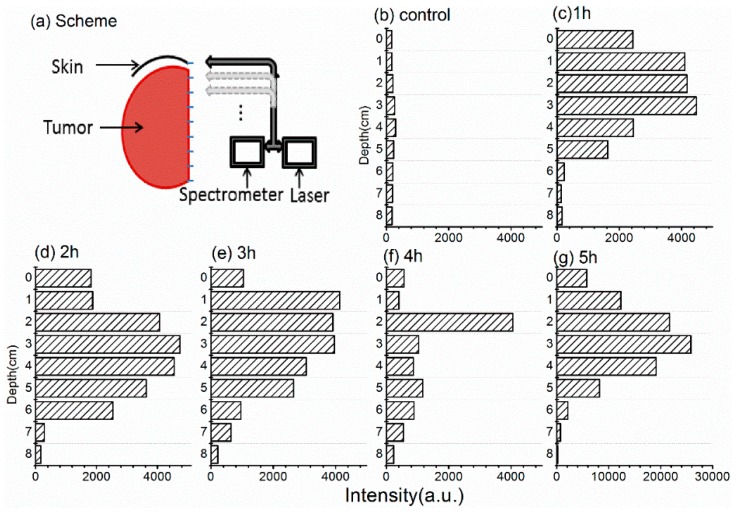
5-Aminolevulinic acid (ALA) penetration into tumors detected by fluorescence spectrometry. (**a**) Schematic showing detection method. Half of a freshly excised tumor with surface skin attached was placed in a holder and scanned for protoporphyrin IX fluorescence using an optical fiber moving along the vertical axis. Fluorescence was recorded at 1-mm intervals; (**b**–**g**) Fluorescence intensity at various depths of the tumor in control mice or detected at 1–5 h after application of ALA hydrogel to the skin. Depth of skin is set at 0 mm. Fluorescence intensity was calculated as described in the methods.

The distribution and concentration of a photosensitizer in the target tumor have important influences on the effect of PDT. For local administration, the depth of photosensitizer penetration directly affects the outcome of PDT treatment; however, it is difficult to measure this in vivo due to the weak fluorescence signal of PpIX and limitation of light penetration in deep tissue [22]. We examined PpIX fluorescence in vertical sections of fresh tumor tissue less than 10 min after excision (as shown in Figure 6a). The tumor and surface skin were dissected from the animal at various times after local administration of ALA hydrogel, and the tumor was cut in half lengthwise. One half was placed in a sample holder and the vertical section was scanned with a fluorescence spectrometer. Measurements were taken at 1-mm intervals and the fluorescence intensity was calculated as described in the methods. The cut-off for positive fluorescence intensity of PpIX was set at 2000 (arbitrary units). The depth of penetration was ~3–6 mm, depending on the time after ALA administration. ALA penetrated to a depth of 3 mm by 1 h and 6 mm by 2 h, but at no time was it detected at depths >6 mm. The fluorescence intensity at 4 h was weak at all levels except 2 mm, which may be due to a greater density of blood vessels in the scanning path. Hemoglobin present in the blood vessels would significantly affect the fluorescence signal.

### 2.6. Distribution of HMME in Tumors

**Figure 7:**
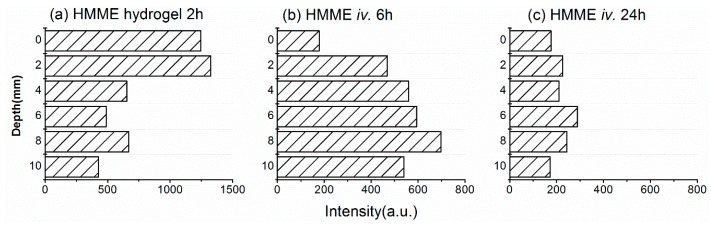
Distribution of hematoporphyrin monomethyl ether (HMME) in tumors measured by fluorescence spectrometry. Tumors were analyzed as described for Figure 5. (**a**–**c**) HMME distribution at 2 h after application of a hydrogel to the skin (**a**) and at 6 h (**b**) and 24 h (**c**) after intravenous administration in phosphate-buffered saline.

We previously noted that local administration in hydrogel form resulted in only low levels of HMME in the subcutaneous tumors (Figure 4b). To confirm this finding, we measured HMME distribution in the tumors using the same spectroscopy method described above for ALA. A substantial HMME fluorescence signal was detected only at depths up to ~2 mm below the skin (Figure 7a), which was consistent with our previous results. We also measured the distribution of HMME in subcutaneous tumors after intravenous administration. In this case, we examined tumors after 6 and 24 h because these were the times of peak HMME uptake in tumor and skin, respectively, in our earlier experiments (Figure 4d). The distribution of HMME in the tumor was relatively uniform at both times, although it was significantly higher at 6 h (Figure 7b,c). However, we did not observe increased HMME levels in the skin at 24 h (Figure 4d), which we speculate is because the skin is too thin for our detection system to pick up the signal precisely.

## 3. Discussion

Various strategies have been employed to enhance topical penetration of ALA or its chemical derivatives and to target the delivery of preformed photosensitizers with relatively high molecular weights, such as porphyrins, chlorins, phthalocyanines, and texaphyrins. With respect to topical penetration, the methods used to enhance the effect of PDT on skin disease and subcutaneous tumors include intratumoral injection, curettage/debulking of nodular lesions, dermabrasion, tape-stripping, sonophoresis, iontophoresis, photomechanical waves, needle-free jet injections, and microneedle arrays [23,24,25,26]. However, few of these methods have been studied for PDT of mucosal or submucosal tumors. Similarly, various carriers and targeting systems have been used to improve delivery of the currently available photosensitizers. Unfortunately, these compounds lack true selectivity for rapidly proliferating cells and are easily degraded in vivo, highlighting the need for more feasible strategies. New photosensitizing molecules and novel modifications of known photosensitizers involve extensive preclinical and clinical evaluation before they can be used clinically. However, innovative formulations for photosensitizer delivery can often be approved in a relatively short time in many countries. In this study, we measured the concentration and distribution of photosensitizers in rectal tumors to explore the feasibility of using hydrogel suppositories containing ALA and HMME for PDT of rectal tumors.

To date, the porphyrin precursor ALA and its methyl ester have been the most widely used agents for topical PDT [27,28]. Topical ALA PDT has shown significant efficacy (response rates ranging from 50% to almost 100%) for several superficial basal cell carcinomas, actinic keratosis, Bowen’s disease, squamous cell carcinoma, as well as non-malignant lesions such as psoriasis, viral warts, papilloma, mycosis fungoides, and other conditions [2]. ALA is a small molecule (167.6 Da), so its diffusion into cutaneous tissue after topical delivery is favorable. However, its hydrophilicity limits its capacity to cross the stratum corneum. Mucosal epithelium has no stratum corneum and ALA should be able to penetrate the tissue with relative ease. In the present study, we showed that PpIX levels in the rectal wall were 9.76- and 5.8-fold that in the skin at 1 and 3 h, respectively, after local administration. This implies that ALA could potentially be delivered as a suppository for the treatment of rectal and anal cancer. In fact, based on the subcutaneous tumor model, we estimated that the level of PpIX in rectal tumors in situ would reach 3–5-fold that observed in skin (Figure 4). The profile of ALA levels in the subcutaneous tumor (Figure 6) indicated a penetration depth of ~3–6 mm at 2 h, and this was not further increased with time. Although ALA penetrates mucosal epithelium more easily than skin, we consider that penetration in rectal tumors would be similar to that in subcutaneous tumors. Thus, hydrogel suppositories are not only suitable for delivery of ALA to the small submucosal tumors in the rectum but also could improve the effect of PDT by increasing the local concentration of ALA.

HMME is a porphyrin derivative and a second-generation photosensitizer produced in China. Preclinical evaluations confirm its efficacy and safety for PDT of port-wine stain birthmarks, and it is currently being developed for broad therapeutic and diagnostic applications [29]. Similar to other porphyrin derivatives, HMME exhibits low permeability of the stratum corneum due to its relatively high molecular weight (>500 Da). To date, there have been no reports on the ability of HMME to penetrate mucosa. In the present work, only low levels of HMME were observed in the rectal wall after local administration via hydrogel suppository, and the HMME fluorescence signal could be measured to a depth of only 2 mm below the skin. These findings suggest that the hydrogel is an inappropriate delivery vehicle for HMME. Because there is a significant difference in the time to peak HMME uptake between the tumor and the skin, intravenous delivery remains a better option for HMME at present.

## 4. Materials and Methods

### 4.1. Materials

5-Aminolevulinic acid hydrochloride (118 mg/vial, powder, purity 99.8%, H20070027) and HMME (100 mg, purity 99.8% , H20120076 ) were purchased from Shanghai Fudan-Zhangjiang Bio-Pharmaceutical Co., Ltd. (Shanghai, China). HMME was dissolved in phosphate-buffered saline (PBS) to generate a 10 mg/mL stock solution stored at −20 °C. Alginate and calcium chloride were purchased from Sigma-Aldrich (St. Louis, MO, USA).

### 4.2. The Formation of Photosensitizer Hydrogels and Release Test in Vitro

A 20 wt % solution of ALA was produced by dissolving 118 mg ALA powder in 500 µL alginate (3%), and a 1-mg/mL solution of HMME was acquired by diluting 50 µL of a 10-mg/mL HMME solution in 500 µL alginate. 50 μL of 20 wt % ALA solution or 1 mg/mL HMME as mentioned above was added into 5 µL 5% CaCl_2_ (the volume ratio was 10:1), then ALA hydrogel would form in around one minute.

The release tests of the PS hydrogel in PBS were performed. In detail, the formed PS hydrogels were placed into 1mL PBS solution with gentle stirring in a 37 °C waterbath. A 100 µL aliquot of the solution was taken out at set time points following the supplement of same volume of PBS. The concentrations of ALA in the gained samples were detected by the typical dimethyamino-benzaldehyde colorimetry while HMME was detected using a fluorescence spectroscope (QuantaMaster 40, Photon Technology International Inc. (PTI), Birmingham, NJ, USA). The release of cumulative percentage to time of both the two PSs were calculated.

The release tests of PS hydrogels in physiological situation were also performed. The formed PS hydrogels were placed in RPMI1640 medium with 10% FBS in which SW837 cells were cultured. The concentrations of the released PS were detected at set time points using the same methods above.

### 4.3. Animal Model

The human rectal adenocarcinoma cell line SW837 was obtained from the Institute of Biochemistry and Cell Biology, Chinese Academy of Sciences (Shanghai, China) and maintained in RPMI 1640 medium supplemented with 10% fetal bovine serum and 1% penicillin-streptomycin solution. SW837 cells (2 × 106) were injected subcutaneously into the right armpit of groups of three BALB/C mice (males, ~4–6 weeks old, ~20 g body weight). Treatment was started when the average tumor size was ~10 mm × 10 mm. The study was approved by the animal welfare committee of institute of biomedical engineering.

### 4.4. Administration of Photosensitizer

In vivo experiments, for intrarectal local administration, 50 μL of 20% ALA or 1 mg/mL HMME was injected into the rectal cavity via the anus while the mouse was held up by the tail to prevent leakage. Gel formation was induced by rapid injection of 5 µL 0.1 M CaCl_2_ into the rectum, and the tail was released to allow formation of the hydrogel suppository.

For cutaneous local administration, 50 μL of 20% ALA or 1 mg/mL HMME was placed on the surface of the skin overlying the subcutaneous tumor, and 5 μL of 0.1 M CaCl_2_ was added to form the hydrogel. The area was covered in plastic wrap to prevent evaporation and the animals were then returned to their cages and allowed to move freely in the dark.

For intratumoral injection, 50 µL HMME (1 mg/mL) diluted in PBS was slowly injected into the middle of the tumor and the needle was then quickly withdrawn. For intravenous delivery, 50 µL HMME (1 mg/mL) diluted in PBS was injected via the tail vein.

### 4.5. Sample Collection

At the indicated times, the animals were sacrificed by decapitation. An area of tissue containing the entire tumor and the surface skin was removed within 10 min of sacrifice, and the samples were cut in half lengthwise. One half was used to determine the photosensitizer distribution as described below. The other half was carefully separated into tumor and skin and was used to determine photosensitizer concentration. A full-thickness section of the rectal wall (~1 cm in length) was dissected above the dentate line.

Samples were collected at 1, 2, 3, 4, and 5 h from animals administered photosensitizer intrarectally, cutaneously, or intratumorally and at 2, 6, 12, 24, and 48 h from animals injected intravenously.

### 4.6. Photosensitizer Concentration Determination

ALA and HMME concentrations in the tissues were determined using a modification of the methods reported by Chen [30]. Standard curves covering a concentration range from 0.01 to 1.56 µg/mL were prepared in mouse plasma. For this, 100 µL of plasma containing 1.7 µg PpIX or HMME was mixed with 1.6 mL 9% NaCl-1M HCl (the 9% NaCl solution contains 1M HCl) and incubated for 10 min. The mixture was centrifuged at 1000× *g* for 10 min and the supernatant (containing 1 µg/mL photosensitizer) was withdrawn and serially diluted 2-fold with 5% NaOH until the concentration was 0.015 µg/mL. The fluorescence intensity of the samples was detected using a fluorescence spectroscope (Quanta Master 40, PTI). The excitation and emission wavelengths for HMME were 395 nm and 627 nm, respectively, and those for PpIX were 395 nm and 638 nm, respectively. Standard curves were constructed with the photosensitizer concentration on the X axis and the fluorescence intensity on the Y axis. The linear equations were calculated by regression.

The excised samples of skin, tumor, and rectal wall were weighed, disrupted in a mechanical homogenizer, and then incubated in a commercial cell lysis buffer to release the photosensitizer. The lysates were centrifuged, and the supernatants were collected and treated in the same manner as described above for the plasma samples used to construct the standard curves. The photosensitizer concentration calculated from the standard curve was divided by the tissue weight to give the photosensitizer tissue concentration, which is expressed as µg/g tissue.

### 4.7. Photosensitizer Distribution in Tumor

**Figure 8:**
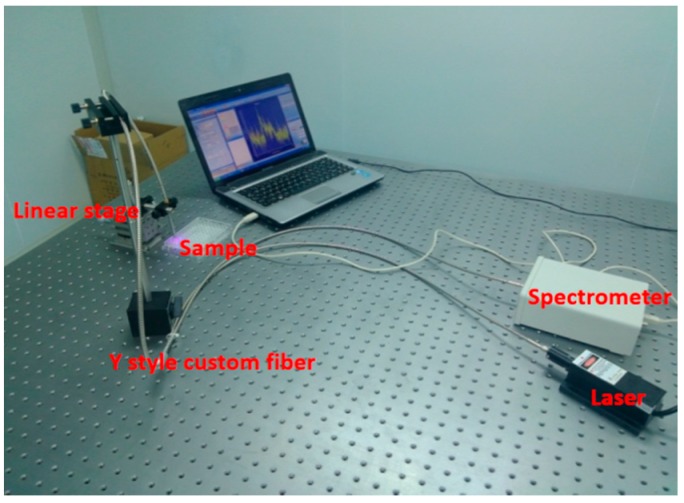
The fluorescence spectrometry system.

The second half of excised tumor sample was placed in a sample holder with the vertical section exposed for detection. The fluorescence spectrometry system was set up in our laboratory and consisted of four parts: a spectrometer (USB4000, Ocean Optics, Dunedin, FL, USA), a light source (USB-ISS-UV-VIS-2, Ocean Optics), a Y-style custom fiber (Ocean Optics), and a linear stage (NFP-1461, Zolix, Beijing, China). The set-up is shown in Figure 8 which works in a similar way as for other studies [31,32,33] The fiber moved along the vertical line of the tissue section from the skin to the tumor, and fluorescence measurements were recorded every 1–2 mm.

Wavelet denoising with soft thresholding was performed to reduce the noise of the fluorescence spectra. The relative photosensitizer concentration was calculated as the peak area between the full width at half maximum to the emission spectrum of the photosensitizer.

### 4.8. Statistical Analysis

Results are presented as means ± standard errors of the mean (SEM). Comparisons between groups were made using oneway ANOVA. *p* < 0.05 was considered statistically significant for all experiments. Statistical analyses were performed using SPSS v13.0 software (SPSS Statistics, Inc., Chicago, IL, USA).

## 5. Conclusions

In conclusion, we found that ALA more easily penetrates the rectal mucosal barrier than the skin, suggesting that local administration as a hydrogel suppository should be considered as a potential ALA delivery route for PDT of rectal cancer. However, the findings in our study will need to be confirmed using a model of rectal tumor in situ, and PDT following ALA delivery by hydrogel suppository should be performed to verify the treatment effects on rectal tumors. Overall, this study provides new insights into photosensitizer delivery for rectal tumors.

## Abbreviations

The following abbreviations are used in this manuscript:

PDT	photodynamic therapy
ALA	5-Aminolevulinic acid
HMME	hematoporphyrin monomethyl ether
PS	photosensitizer
CRC	colorectal cancer
*iv.*	intravenous
PpIX	protoporphyrin IX
PBS	phosphate-buffered saline
FBS	fetal bovine serum
